# The efficiency and safety of temozolomide and PD-1/L1 inhibitors in pretreated NSCLC with brain metastasis: a retrospective cohort

**DOI:** 10.1007/s00432-024-05808-0

**Published:** 2024-05-23

**Authors:** Xiaobing Li, De Wu, Jing Tang, Yuebing Wu

**Affiliations:** 1 Department of Thoracic Oncology, Hubei Cancer Hospital, Tongji Medical College, Huazhong University of Science and Technology, Wuhan, China; 2 The Centre of Molecular Diagnosis, Hubei Cancer Hospital, Tongji Medical College, Huazhong University of Science and Technology, Wuhan, China; 3 Department of Lymphoma, Hubei Cancer Hospital, Tongji Medical College, Huazhong University of Science and Technology, Wuhan, China

**Keywords:** Temozolomide, PD-1/L1, NSCLC, Brain metastasis

## Abstract

**Objective:**

Previous research has shown that both temozolomide (TMZ) and PD-1/L1 inhibitors (PD-1/L1) alone exhibit certain potential in the treatment of non-small cell lung cancer (NSCLC) with brain metastases (BM), in this study, we will explore combining the two in order to seek new effective treatment options for NSCLC with BM.

**Material and methods:**

During 2021.1 to 2023.12, we collected the date of these pretreated-NSCLC with BM who accept the treatment of TMZ and PD-1/L1, the objective response ratio (ORR), progression-free survival (PFS) and overall survival (OS) were set as the primary endpoint, meanwhile, the toxicity of such regimen was also recorded.

**Results:**

About 42 patients are enrolled, our primary analysis demonstrated that the ORR of such regimen toward NSCLC with BM was 26.19%, with Approximate intracranial and extracranial lesion ORR was 6% and 20% respectively, the DCR was about 64.29%, the mean PFS and OS was about 4 m and 8.5 m. Further analysis indicated that the efficiency correlated with the diagnosis-Specific Graded Prognostic Assessment (ds-GPA) score. Moreover, the toxicity can also be tolerated, indicating the application potential of such regimen against NSCLC with BM.

**Conclusions:**

Our results exhibited that with tolerated toxicity, the combination of TMZ and PD-1/L1 shows promising efficiency against NSCLC with BM, this would be of great significance for the treatment of NSCLC with brain metastasis. However, due to the limitation of sample and retrospective property, the real value of such regimen needed to be further confirmed in the future.

## Introduction

Brain metastasis was a common metastasis site of non-small cell lung cancer (NSCLC). Although radiotherapy (RT) was the main treatment modality for NSCLC with BM, it lacks of long term efficiency, meanwhile, the neurocognitive toxicity is also unavoidable, indicating that new mean is urgently needed in order to improve the prognosis of NSCLC with BM (Suh et al. 2020).

During the past decades, immunotherapy exemplified by targeting PD-1/L1 had greatly changed the landscape of advanced NSCLC (Garassino et al. 2023; Novello et al. 2023), however, there still lack of high-grade evidence of immunotherapy toward the population of brain metastasis (Barlesi and Tomasini 2020). Since most of this evidence result from PD-1/L1 alone (Dudnik et al. 2016; Gadgeel et al. 2019), in order to improve the efficiency, it is rather necessary to adopt the strategy of drug combination (Eguren-Santamaria et al. 2020), such as PD-1 plus CTLA-4 (Brahmer et al. 2023), PD-1 plus CT (Powell et al. 2021), etc. Although there may exist multiple possibility to exert drug combination strategies, from the perspective of clinic feasibility, PD-1 plus CT would be a mean with Cost-effectiveness (Rosell and Karachaliou 2016). Although the efficiency of PD-1 and CT had been widely reported both in the first line and the second line treatment toward the general population of NSCLC, however, the date against special population such as brain metastasis is rather scarce (Loiola et al. 2021). One possible explanation lies in the Uncertainty of such strategy, another would be the exploration of optimal partner for drug combination (Zheng et al. 2017; Henon et al. 2020; Heinhuis et al. 2019). Under such circumstances, with relatively higher capacity penetrating blood–brain barrier (BBB), TMZ would an ideal partner for PD-1/L1 combination, especially for NSCLC with brain metastasis (Cortot et al. 2006; Giorgio et al. 2005; Robins et al. 2006; Yang et al. 2020). Actually, latest case report indeed exhibited that the drug combination of PD-1 and TMZ could bring about long term survival benefits for NSCLC with BM (Zhu et al. 2022), indicating the potential synergism between PD-1 and TMZ, however, up to now, no study about the drug combination of PD-1 and TMZ had been reported in NSCLC with BM, therefore, in this study, we retrospectively analyzed the safety and efficiency of TMZ and PD-1 against pre-treated NSCLC suffering from BM with the aim to seek new way to improve the therapeutic efficiency of such special population.

## Materials and methods

### Inclusion and exclusion criteria

Patients between the ages of 18 and 70 diagnosed with advanced NSCLC, confirmed clinically and pathologically, were eligible for enrollment. Inclusion criteria encompassed individuals exhibiting drug resistance or intolerance to a minimum of two prior chemotherapeutic regimens, including platinum-containing regimens and EGFR TKI therapy. However, patient’s intolerant to second-line chemotherapy alone were also considered for inclusion due to limited therapeutic alternatives. Additional enrollment prerequisites comprised an Eastern Cooperative Oncology Group (ECOG) performance status (PS) of 0 or 1, at least one measurable lesion defined by RECIST criteria, and satisfactory hematologic, hepatic, and renal function. Prior to enrollment, patients had not undergone immune checkpoint therapy, such as anti-PD-1 or anti-CTLA-4. Exclusion criteria comprised a history of hemoptysis (defined as one-half teaspoon of bright red blood within 3 months prior to enrollment), tumors invading major blood vessels, asymptomatic CNS metastases, and uncontrolled hypertension. Current or recent utilization of full-dose anticoagulants or thrombolytic agents for therapeutic purposes was prohibited, although prophylactic use of anticoagulants was permissible. Criteria for transitioning to third-line therapy (including chemotherapy or targeted therapy) relied on evaluation via computed tomography (CT) and magnetic resonance imaging (MRI). For patients diagnosed with central nervous system metastases, the diagnosis-Specific Graded Prognostic Assessment (ds-GPA) score at the initiation of regimen treatment was also documented. The ds-GPA scores were categorized according to this paper’s classification, ranging from 0 to 1 (worst prognostic group), 1.5 to 2.5, 3, to 3.5 to 4 (best group).

### Gene detection

Needle aspiration guided by CT was predominantly utilized for diagnostic purposes among the patient cohort. Following pathological confirmation of diagnosis, a portion of the sample underwent analysis via multiple gene detection methods, including EGFR, ALK, C-Met, and K-RAS. Standard assays were employed for the detection process.

### Drug application

All patients received two or more lines of treatment. Upon clinical confirmation of disease progression or drug resistance, the treatment regimen comprised the following: intravenous administration of temozolomide at a dosage of 150 mg/m^2^, and fixed dosages of PD-1/L1 inhibitors (such as camrelizumab, sintilimab, toripalimab, tislelizumab, pembrolizumab, nivolumab, atezolizumab, among others), typically administered at 200 mg each time, in accordance with their respective prescription instructions. Both drugs were administered intravenously once every 3 weeks, with each cycle lasting 21 days.

### Efficiency evaluation

All patients underwent at least one cycle of treatment. Evaluation of treatment efficacy occurred every 4 weeks through lung CT and brain MRI scans. The primary criterion for evaluation was RECIST 1.0.

### Statistical analysis

All analyses were conducted using SPSS 13.0. Progression-free survival (PFS) was defined as the duration from the initial administration of the TMZ plus PD-1/L1 regimen to the date of confirmed disease progression or death. Overall survival (OS) was defined as the period from the first administration of the TMZ plus PD-1/L1 regimen to death. Patients with unavailable information regarding death or disease progression had their data censored at the date of the last assessment. Descriptive summaries of PFS and OS were provided, along with two-sided 95% confidence intervals (CIs). PFS and OS were estimated using the Kaplan–Meier method, and corresponding figures were generated using Graph Prism 5.0. Statistical significance was assumed at a P-value of less than 0.05.

## Results

### Patient characters

In our study, a total of 42 patients with advanced NSCLC suffering from BM were included, all of whom had undergone treatment with two or more lines of therapy. The predominant pathological type was adenocarcinoma, followed by lung squamous cell carcinoma. Approximately 60% of the patients presented with measurable brain metastases (BM) detectable by MRI or CT scanning. The ECOG PS of the patients ranged between 0 and 2. Among the 42 patients, 18 were female and 24 were male, with an average age of 66 years. The majority of patients had wild-type EGFR status; however, three patients with EGFR mutations experienced disease progression following first-line TKI treatment. Notably, most male patients had a history of heavy smoking, whereas female patients were predominantly non-smokers (refer to Table 1).

**Table 1:** Baseline clinical characteristics of the study cohort

Characteristics	No. of patients (%)
Age	
Years	66
Range	47–75
Gender	
Male	24(57.14%)
Female	18(42.86%)
Smoking history	
Never smoker	14(33.33%)
Former smoker	28(66.67%)
Histology	
Adenocarcinoma	26(61.90%)
Squamous carcinoma	16(38.10%)
ECOG score	
0–1	31(73.81%)
≥ 2	11(26.19%)
GPA score	
0–1	6(14.29%)
1.5–2.0	10(23.81%)
2.5–3.0	11(26.19%)
3.5–4.0	15(35.71%)
PD-L1 expression level	
< 1%	21(50.00%)
1–49%	12(28.57%)
>> 50%	9(21.43%)
Previous radiotherapy	
Yes	13(30.95%)
No	29(69.05%)
Brain metastasis	
Measurable	11(26.19%)
Unmeasurable	31(73.81%)
Bone metastasis	
Yes	25(59.52%)
No	17(40.48%)
Liver metastasis	
Yes	7(16.67%)
No	35(83.33%)
Stage	
IVA	6(14.29%)
IVB	17(40.48%)
IVC	19(45.24%)

### Previous treatment

Most patients with EGFR wild-type status had undergone treatment with two or more lines of chemotherapy. The first-line chemotherapy regimen consisted of pemetrexed plus platinum, while docetaxel or gemcitabine were preferred options for second-line therapy. Approximately 30.95% of these patients had previously received whole-brain RT, with a minimum interval of three months before enrollment. All patients with EGFR mutations had received first-line treatment with a tyrosine kinase inhibitor (TKI), including icotinib, erlotinib, or gefitinib. The specific gene mutations observed in these patients included Exon 19 deletion (1 patient) and Exon 21 L858R mutations (2 patients). Following disease progression, additional genetic testing was conducted for all these patients. One patient with the T790M mutation received direct treatment with Osimertinib, while the other two patients opted for chemotherapy, with regimens similar to those used for patients with EGFR wild-type status (refer to Table 1).

### Efficiency

**Fig. 1:**
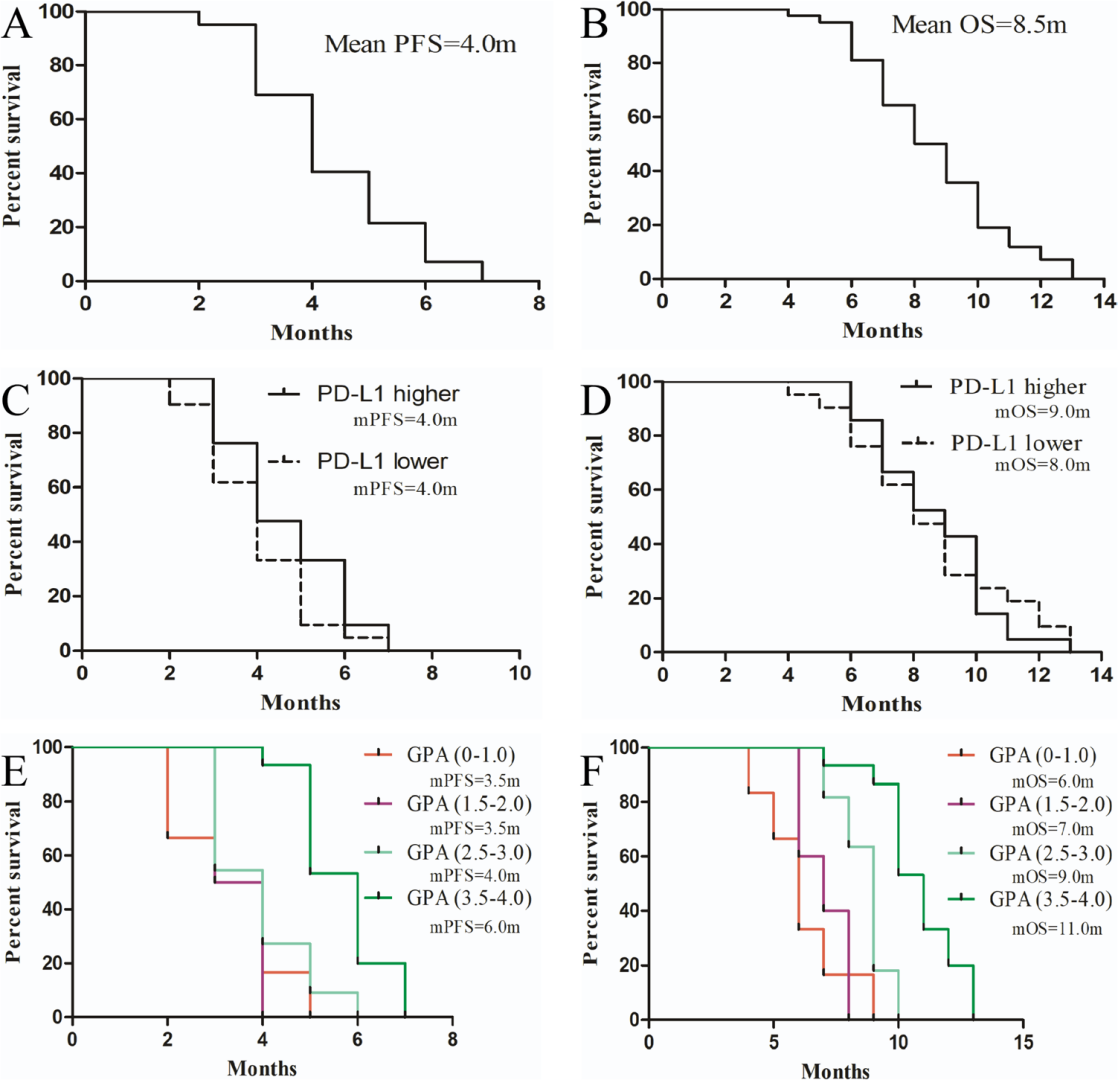
PFS and OS analysis of general population and subgroup patients with PD-1/L1-treated advanced NSCLC who accepted the drug combination of TMZ and PD-1/L1 inhibitors in this study. **A, B** The overall PFS and OS in this study. **C, D** Comparisons of PFS and OS between these patients with higher expression of PD-L1 and lower expression of PD-L1. **E, F** Comparisons of PFS and OS between these patients according to different Ds-GPA categorization. Notes: mPFS, median progression-free survival; mOS, median overall survival; PD-L1, programmed death ligand 1; NSCLC, non-small cell lung cancer; TMZ, temozolomide; Ds-GPA, Diagnosis-specified Graded Prognosis Assessment

All patients underwent a minimum of one cycle of treatment, with a mean treatment line of three cycles for the combination therapy of TMZ plus PD-1/L1. Our efficacy analysis revealed that none of the patients achieved complete response (CR), while 11 patients showed partial response (PR), 16 patients had stable disease (SD), and 15 patients experienced disease progression. The objective response ratio (ORR) of this regimen for NSCLC with BM was 26.19%. The approximate intracranial and extracranial lesion ORRs were 6% and 20%, respectively. The mean PFS was 4.0 months, with a disease control rate (DCR) of approximately 64.29%. The median OS was 8.5 months (refer to Table 2 and Fig. 1A and B).

**Table 2:** Clinical activity of TMZ plus PD-1/L1 inhibitors in advanced NSCLC with brain metastasis

	Patient no	Ratio
Complete response	0	0
Partial response	11	26.19% (11/42)
Stable response	16	38.10% (16/42)
Progressive disease	15	35.71% (15/42)
Objective response		26.19%
Median PFS		4.0 m
Disease control rate		64.29%
Median OS		8.5 m

### Biomarker exploration

To further explore potential priority populations for this regimen, subgroup analyses were conducted. Preliminary findings indicated that there was no significant difference in efficacy between patients with PD-L1 expression levels ≥ 1% and those with < 1% (refer to Fig. 1C and D). However, a notable correlation emerged between the efficacy of this regimen and the ds-GPA score—a recognized prognostic scoring model for lung cancer BM, considering PS score, age, number of BM, presence of intracranial symptoms, etc. Lower scores were associated with better efficacy, suggesting that the ds-GPA score could serve as a potential marker for selecting priority populations for this regimen (refer to Fig. 1E and F).

### Toxicity

During the treatment, common treatment-related hematological adverse events (AEs) included anemia, leukopenia, thrombocytopenia, alopecia, nausea, vomiting, rash, hyperthyroidism, hypothyroidism, and others. Most of these side effects were tolerable, with severity graded as one or two. The mean dosage of TMZ was 200 mg. AEs of grade three or four included anemia, leukopenia, thrombocytopenia, neutropenia, nausea, abnormal liver function, and others. The discontinuation rate of the drug for patients experiencing these side effects was 12%. Serious AEs could be mitigated or reduced by implementing corresponding supportive therapies (refer to Table 3).

**Table 3:** Adverse events of TMZ plus PD-1/L1 inhibitors in advanced NSCLC with brain metastasis

Adverse event	TMZ plus PD-1/L1 inhibitors [n (%)]	
	Any grade	Grade 3 or 4
Anemia	11(26.19%)	3(7.14%)
Leukopenia	6(14.29%)	4(9.52%)
Thrombocytopenia	8(19.05%)	3(7.14%)
Neutropenia	5(11.90%)	3(7.14%)
Fatigue	7(16.67%)	2(4.76%)
Alopecia	8(19.05%)	2(4.76%)
Nausea	9(21.43%)	3(7.14%)
Vomiting	8(19.05%)	2(4.76%)
Rash	9(21.43%)	1(2.38%)
Pruritus	7(16.67%)	2(4.76%)
Hyperthyroidism	8(19.05%)	1(2.38%)
Hypothyroidism	9(21.43%)	1(2.38%)
Abnormal liver function	6(14.29%)	3(7.14%)
Diarrhea	5(11.90%)	2(4.76%)
ILD	4(9.52%)	1(2.38%)

## Discussions

In recent years, immune checkpoint therapy represented by PD-1/L1 has significantly transformed clinical practice for advanced lung cancer. Although immunotherapy can provide long-term survival benefits for some advanced lung cancer patients, there still exist room for efficacy improvement, as only a minority (just 20%) of patients derive benefit from it (Garassino et al. 2023; Novello et al. 2023). In addition to identifying reliable predictive markers, another practical strategy to enhance the effectiveness of immunotherapy is through combination therapy, especially for specific subgroups such as those with liver metastasis, brain metastasis, pleural effusion, or co-mutations (Rosell and Karachaliou 2016; Henon et al. 2020). For lung cancer with brain metastasis, besides combining conventional treatment modality such as RT (Minniti et al. 2014), exploring other combination regimens such as combining chemotherapy (Powell et al. 2021) or dual immunotherapy (Brahmer et al. 2023; Paz-Ares et al. 2021) are also of great significance. Although dual immune therapy models exemplified by Checkmate-227 and Checkmate-9LA have shown certain efficacy against brain metastasis in advanced NSCLC, the proportion of patients with brain metastasis in these trials is low, and dual immunotherapy still faces challenges such as severe adverse reactions and high treatment costs in clinical practice (Uprety et al. 2024). Therefore, from a clinical feasibility perspective, combining chemotherapy with PD-1 for the treatment of brain metastasis is likely to be a cost-effective choice (Heinhuis et al. 2019; He et al. 2017).

Unfortunately, existing chemotherapy drugs not only have limited efficacy against brain metastasis, but also there is a lack of data on the use of chemotherapy alone or in combination with PD-1 for brain metastasis treatment. Considering that immunotherapy combined with chemotherapy has gradually become the mainstream treatment mode in clinical application of immunotherapy, prioritizing chemotherapy drugs with strong BBB penetration for combination with immunotherapy in the treatment of NSCLC brain metastasis seems reasonable (Zheng et al. 2017; Heinhuis et al. 2019). In fact, some case reports and small-sample retrospective analyses have indeed shown the potential of temozolomide combined with immunotherapy in treating lung cancer with brain metastasis (Yang et al. 2020; Zhu et al. 2022).

As for temozolomide, although there are many reports of its use in the treatment of lung cancer brain metastasis, most of them are in combination with RT, and there are also issues such as inconsistent reported efficacy and severe adverse reactions (Cortot et al. 2006; Addeo et al. 2008; Sperduto et al. 2013). Therefore, combining PD-1 with temozolomide not only explores new combination therapies but also opens up new avenues for the treatment of solid tumor with brain metastasis, including NSCLC. Consistent with our expectation, the drug combination of PD-1 and TMZ indeed bring about promising efficiency, with overall ORR, mean PFS and OS was 26%, 4 m and 8.5 m. Besides promising efficiency, the toxicity of such regimen is also low. The AEs ratio of 3 or above was about 30%. All of this date indicated the potential of such regimen for the treatment of NSCLC with brain metastasis. In this study, besides confirming the preliminary efficacy and safety of the regimen in the treatment of lung cancer BM, we have also introduced some additional innovations. For instance, in terms of treatment protocol, in order to reduce toxic side effects, we adjusted the dosage and administration of temozolomide from the standard 200 mg/m^2^ continuous oral administration for 5 days (Giorgio et al. 2005; Robins et al. 2006; Duan et al. 2020) to 150 mg/m^2^ every 3 weeks intravenous infusion. This adjustment not only did not affect efficacy but also significantly reduced temozolomide-related toxic side effects. Moreover, patient compliance with treatment significantly improved. This exploration was driven by the idea that when chemotherapy drugs are combined with immunotherapy, low doses of chemotherapy drugs may be sufficient to induce immunogenic cell death (ICD) in tumor cells, thereby stimulating tumor antigen release to activate the immune system and exert a synergistic therapeutic effect (Zheng et al. 2017; Heinhuis et al. 2019). Furthermore, we found that although PD-L1 expression levels cannot predict the efficacy of this regimen, which may be related to the small sample size and the complex microenvironment of BM (Goldberg et al. 2016), we unexpectedly discovered a close correlation between the Ds-GPA score and the efficacy of the regimen (Sperduto et al. 2010). As far as we know, this is a new discovery which has not been reported in previous studies, suggesting that composite indices represented by Ds-GPA may hold potential for predicting the efficacy of immunotherapy in the treatment of BM, rather than being limited to single indicators such as PS score, PD-L1 expression levels, tumor burden, etc. (Hendriks et al. 2019; Hulsbergen et al. 2020). Finally, all of these evidences not only provide new options for future immune combination therapies but also offer new insights and directions for the treatment of specific populations with immunotherapy (Barlesi and Tomasini 2020; Jindal and Gupta 2018).

To sum up, as far as we know, this is the first study to report the promising efficiency of PD-1 and TMZ in NSCLC with brain metastasis together with lower cytotoxicity, further study exhibited that the efficiency of such regimen correlate with Ds-GPA score, indicating that Ds-GPA score can be utilized as a potential parameter for efficiency prediction of such regime (Sperduto et al. 2010). Although the preliminary results are promising, there still exist some problems needed to be resolved in the future, such as the accurate biomarker for efficiency prediction, the dosage optimization of this regimen, the elucidation of synergistic mechanism, etc. (Rosell and Karachaliou 2016; Henon et al. 2020; Jindal and Gupta 2018). Only these problems are resolved gradually, the efficiency of such regimen can be maximized, and more and more patients would benefit (Henon et al. 2020).

## Conclusions

This study has confirmed that the combination of TMZ and PD-/L1 inhibitors has lower toxic side effects and promising therapeutic efficacy for the treatment of NSCLC with brain metastasis. It has also further identified the potential parameter for efficiency prediction and modify the regimen. However, from the perspective of truly changing the treatment paradigm, the clinical value of this regimen still requires further research and validation.

## Key findings

This study marks the first documentation of the promising efficacy of combining temozolomide and PD-1/L1 inhibitors, demonstrating tolerable toxicity levels, in treating advanced NSCLC patients with brain metastasis. The efficacy appears to correlate with the ds-GPA score of patients.

## What is known and what is new?

Historically, both temozolomide and PD-1/L1 inhibitors have exhibited limited efficacy in addressing brain metastases from advanced NSCLC.

However, the concurrent administration of temozolomide and PD-1/L1 inhibitors reveals a potential synergistic effect against advanced NSCLC patients with brain metastasis.

## What is the implication, and what should change now?

The findings suggest that this combined regimen holds promise as a viable treatment option for NSCLC with brain metastasis. However, further confirmation of its true value is imperative through continued research and clinical trials.

## Data Availability

No datasets were generated or analysed during the current study.
